# Characterization of the Quality and Oxidative Stability of Hemp-Oil-Based Oleogels as an Animal Fat Substitute for Meat Patties

**DOI:** 10.3390/foods11244030

**Published:** 2022-12-14

**Authors:** Irfan Hamidioglu, Gitana Alenčikienė, Miglė Dzedulionytė, Aelita Zabulionė, Aditya Bali, Alvija Šalaševičienė

**Affiliations:** Food Institute, Kaunas University of Technology, Radvilėnų 19 C, LT-50254 Kaunas, Lithuania

**Keywords:** hemp oil, waxes, oleogel, chemical stability, pork fat substitutes

## Abstract

The effect of the incorporation of rice bran wax (5%; 7%) or candelilla wax (3%; 7%) for production of hemp-oil-based oleogels was analyzed in this study. The experiment was carried out to replace between 0 and 100% of animal fat in meat patties with oleogels. Free fatty acids (FFAs), acid value (AV), oxidative stability index (OSI), conjugated diene value, malondialdehyde value, physicochemical properties, and the sensory properties of oleogels and meat patties were studied. The results indicated that hemp oil had more polyunsaturated fatty acids and lower oxidative stability when compared to oleogels. The OSI for oil was 3.1 h, while for oleogels it was 3.4–3.6 (candelilla case) or 3.7–3.9 (rice bran). Oleogels were able to match pork fat texture properties such as spreadability and adhesiveness in meat patties. However, sensory data for cooked meat patties with animal fat fully replaced by oleogels revealed that samples with 100% pork fat had higher juiciness and taste intensity. Our results showed that a wax-based oleogel had a higher oxidative stability and nutritional profile, but further investigations to mimic pork fat properties in meat patties are necessary.

## 1. Introduction

Fats and oils are essential for human nutrition, metabolism, and the sensory palatability of foods. According to WHO recommendations, the daily total energy intake of oils and fats for the adult human should not exceed 30% and saturated fats should constitute less than 10% of the total energy intake [1,2]. Dietary recommendations suggest decreasing saturated fats in the diet and shifting to unsaturated fats. However, such a change is difficult for the food industry as saturated fatty acids provide important physicochemical properties which are responsible for the texture and taste of products, which are important properties for consumers [3]. At the same time, saturated fatty acids demonstrate a higher stability against oxidation processes than polyunsaturated fatty acids. The direct replacement of saturated fats with oils is challenging as oils are in liquid phase at room temperatures and have high hydrophobicity, which can lead to phase separations in food products.

Oleogelation is considered as a possible solution [4] to convert liquid vegetable oil into a solid-like phase. Suitable oleogelators, usually low-molecular-weight molecules (such as plant waxes and certain proteins), are added to the continuous liquid phase at small concentrations. Oleogelators form a stable, solid network which immobilizes liquid oils.

Currently, oleogels are considered suitable alternatives to saturated fatty-acid-containing fats for use in foods. They provide the nutritional advantage of oils such as polyunsaturated fatty acids in addition to having the favorable technological and sensory characteristics of saturated fats, such as hardness [5].

Different waxes such as candelilla wax, rice bran wax, beeswax, carnauba wax, and sunflower wax can be used as oleogelators [6]. Waxes have numerous advantages over other materials, such as being food-grade and having a low cost, good availability, and excellent gelling ability. Most waxes consist of long-chained saturated fatty acids, usually C-20 to C-26, and fatty alcohols with long chain lengths. Oleogels produced with rice bran and candelilla waxes can maintain stability of structure despite their low solid fat content.

An amount of 1–3% w/w of candelilla wax is enough to form an oleogel, however the structuring ability of this wax could be decreased during physical treatment following the separation of oil [7]. Higher amounts of candelilla wax increase the stability of oleogels in general. However, the most significant changes in oleogel stability are determined when concentrations of candelilla wax are changed from 3% to 7% [8].

Candelilla and rice bran waxes as oleogelators have been analyzed in various vegetable oils such as sunflower oil [9,10], canola oil [11], olive oil [12], and others. They have also demonstrated good potential for use in food [13], cosmetics, pharmaceutical products, and polymers.

The kinetic stability and rheological properties of the oleogels depend on the type of vegetable oil used. Oleogels can be developed from numerous vegetable oils, including linseed, pumpkin seed, and hemp seed oil [14]. These oils are a rich source of essential fatty acids and have a multitude of health benefits such as decreased inflammation, lowered risk of heart disease, and cognitive enhancement. Consequently, the consumption of Omega 3-fortified functional foods has increased in recent decades.

Processed meat products including patties are among the most-consumed meat products in many countries. The consumption of processed meat products has become increasingly popular owing to their delightful flavor, cost-effectiveness, and the variety of product opportunities that arise from using different types of fat. Today, consumers demand natural and healthy food products with improved nutritional features [15]. Therefore, improving meat products by reducing the fat grade has become imperative for producers.

It is assumed that oleogels developed using waxes obtained from candelilla, rice bran, and various vegetable oils will affect meat patties’ properties. Therefore, we hypothesized that (1) it is possible to develop stable oleogels by structuring hemp oil with candelilla or rice bran waxes and (2) the technological–functional properties of such oleogels will be similar to those of animal fat and this will allow for the use of oleogels as an animal fat replacement in meat patties.

The objective of this study was to characterize the oxidative stability, processing features, lipid oxidation, texture, and sensory properties of oleogels prepared for use as animal fat substitutes for meat patties.

## 2. Materials and Methods

### 2.1. Materials

Fresh, legal-grade hemp (*Cannabis sativa* L.) seeds were purchased from the Lithuanian local company Allive Europe JSC (Lithuania) and stored at 4 °C until use. Rice bran and candelilla waxes were obtained from WARUM Ulrich GmbH (Eresing, Germany). Fresh, lean beef and pork back fat were obtained from a local Lithuanian market. Fatty acid methyl ester (FAME) standards were obtained from Toronto Research Chemicals (Toronto North York, ON, Canada). Sodium thiosulfate, 2,2,4-trimethylpentane (isooctane), starch solution, potassium iodide, acetic acid, glacial, phenolphthalein, sodium hydroxide 0.1 N, ethyl alcohol, diethyl ether, and P-anisidine (para-anisidine) were purchased from Sigma–Aldrich (St. Louis, MS, USA).

### 2.2. Preparation of Oleogels

Locally procured hemp seeds were cleaned and sorted, after which they were cold-pressed using laboratory-scale cold press equipment (MLVS-04, Lithuania). The oil temperature after pressing was approximately 30–35 °C. The oil was then filtered and centrifuged for approximately 10 min at 2800 rcf. After filtration, the oil (purified Hemp oil) was collected and stored at 4 °C for further use.

Oleogels were produced from the purified hemp oil by mixing it with rice bran wax or candelilla wax [16]. Hemp-seed-oil oleogels were prepared at two different concentrations, as determined during preliminary experiments: candelilla-wax oleogels were developed at 3% *w*/*w* candelilla wax concentration (O-C3) and at 7% *w*/*w* candelilla wax concentration (O-C7). Similarly, rice-bran-wax oleogels were developed at two different concentrations, specifically 5% *w*/*w* (O-R5) and 7% *w*/*w* (O-R7). Hemp oil was used as a control. To summarize the process, hemp oil was mixed with the concentrations of waxes mentioned above, and the prepared samples were stirred for 8 min at 85 °C before being removed from the bath and cooled. Samples were then stored at 4 °C for approximately 1 h to increase the structural stability of the oleogels, after which the samples were stored at 25 °C temperature for 55 days for the analysis of the oxidation process and at +4 °C for the preparation of meat patties.

### 2.3. Manufacture of Meat Patties

Lean beef meat (*M. Semimembranosus*, moisture: 75.12% ± 0.06; protein: 19.32% ± 0.53; fat: 2.82 ± 0.35) and pork back fat (moisture: 11.42% ± 0.24; protein: 8.32% ± 0.10; fat: 78.94 ± 0.38) were obtained from a local meat market. Beef, oleogels, and pork back fat were ground separately on a plate with 3 mm orifices and stored at 4℃ until the patties were manufactured. The control meat patties were prepared according to the following formulation: beef (67%), pork back fat (20%), ice (12%), and sodium chloride (1%). The temperature of the meat batter did not exceed 12 °C during homogenization. Five treatments were prepared: meat patties with pork back fat were used as a control, and in the other formulations pork fat was replaced with the prepared oleogels. After homogenization, the meat patties were shaped into discs (Æ10 cm × 1 cm). The control patty MP-C, meat patty MP-OR5 (oleogel containing 5% rice bran wax), meat patty MP-OR7 (oleogel with 7% rice bran wax), meat patty MP-OC3 (oleogel with 3% candelilla wax) and meat patty MP-OC7 (oleogel with 7% candelilla wax) were stored in a refrigerator at 1.5 °C on a tray lined with wax paper to prevent drying.

### 2.4. Cooking of the Meat Patties

Prior to analysis, the prepared meat patties were cooked in a convection oven at 180 °C for 20 min, which was sufficient to produce an internal temperature >75 °C. The cooked patties were taken out of the oven, cut into quarters, and wrapped in aluminum foil, after which they were placed in labeled trays and put back into the oven at 60 °C within 4–5 min from preparation until they were served to panelists for sensory analysis.

### 2.5. Methods of Analysis

#### 2.5.1. Proximate Analysis

The chemical compositions of the oils, oleogels, uncooked and cooked meat, and the meat patties—including moisture, fat, and protein—were determined according to appropriate specific official methods of analysis [17].

#### 2.5.2. Oxidative Stability of Lipids

Peroxide value (PV) was determined according to ISO 3960:2010 [18]. The free fatty acids (FFAs) and acid values (AVs) of the oleogel samples were determined according to AACC [19] (2009) with slight modifications. FFA content was defined as the amount of potassium hydroxide (in milligrams) required to neutralize the FFA present in 1 g of fat. The acidic content of FFAs was determined as follows: approximately 5 g of a sample was weighed and placed in a water bath at 60 °C (10–20 s) The sample was mixed with ethanol (96%) and diethyl (peroxide-free) ether at a 1:1 ratio. Phenolphthalein was added to the mixture. Finally, the mixture was titrated with 0.1 M sodium hydroxide. The results were expressed as oleic acid (282 g/mol) for the FFA and in milligrams per gram for the acid value. FFA and AV values were measured during the storage of the oleogels at 1, 6, 12, 20, 33, and 50 days.

#### 2.5.3. Oxidative Stability Index (OSI)

Rancimat analysis was performed using Rancimat 892 (Metrohm, Herisau, Switzerland) according to the protocol described by Läubli and Bruttel [20]. Three grams of the oleogel samples were placed into test tubes. Oils with no added wax were used as blank control samples. The oleogel samples were carefully placed into each reaction vessel to avoid pushing the substance into an oxygen glass tube. The temperature was set to 120 °C and the airflow rate was fixed at 20 L/h. The induction period was automatically determined from the inflection point of the curve using software supplied by the company. Results were expressed as the induction period (IP) time (h) of the samples.

#### 2.5.4. Fatty Acid Composition

Fatty-acid-composition analysis was based on ISO 12966-2:2017 [21] with slight modifications. The fatty acid composition was investigated using an Agilent 7890 gas chromatograph coupled with an Agilent 5975 mass spectrometer (GC–MS; Agilent Technology, Santa Clara, CA, USA). The setup was equipped with an HP-88 capillary column (100 mm × 0.25 mm id, 0.2 m film thickness). To prepare fatty acid methyl esters (FAME), oleogels were saponified with 0.5 M of Potassium hydroxide and then methylated using 40% boron trifluoride in methanol. The injection temperature was 250 °C and the split ratio was set to 1:30. Helium was used as a carrier gas at a pressure of 100 kPa. The process began with 5 min at 80 °C, then was increased up to 150 °C at 10 °C/min and 2 min at 150 °C, followed by another increase up to 230 °C at 5 °C/min and 10 min at 230 °C. Oven temperature was programmed in accordance with the following sequence. The ionization voltage was 70 kV and the scanning range was 50–550 m/z.

#### 2.5.5. Conjugated Diene Value

Conjugated diene value (CDV) was determined according to White [22]. The oleogel sample (0.01 g) was weighed carefully into a 25 mL volumetric flask. The sample was dissolved in iso-octane (2, 2, 4-Trimethylpentane) and gently mixed. The absorbance of the sample was measured at 233 nm using an Agilent 5975 mass spectrometer (GC–MS; Agilent Technology, CA, USA). The blank sample with iso-octane was measured using a quartz cuvette. The absorbance of the dissolved samples was measured using a quartz cuvette.

#### 2.5.6. Malondialdehyde Value

A quantitative analysis of malondialdehyde (MDA) in untreated patties was carried out according to the thiobarbituric acid (TBA) assay using a Cary 60 UV-VIS spectrophotometer (Agilent Technologies). MDA is detected as the product of lipid peroxidation, which reacts with thiobarbituric acid leading to a change in the color of the solution from transparent to red. The procedure for the determination of MDA [23] is described as follows: for the TBA stock solution, 15 g of trichloroacetic acid, 0.375 g of thiobarbituric acid (0.375% *w*/*v*), and hydrochloric acid (0.25 N) were added to a flask of 100 mL and diluted to 100 mL with water. The solution was then heated to improve the dissolution of the thiobarbituric acid. An amount of 0.5 g of the sample was weighed in a test tube, and TBA (2.5 mL) was added to the sample and boiled for 10 min until the color became pink. Following this, the tubes were cooled under cold water, the sample was placed for 30 min in a sonicated bath, and later the sample was centrifuged at 5000× *g* (10 min). The absorbance of the retentate was measured at 535 nm against the blank (TBA). The calibration curve was set on a UV-VIS spectrophotometer with an MDA concentration within a 1–10 mg/L range and the results were quantified as malondialdehyde equivalents (mg MDA/kg sample). Samples were tested at 2,4, 6, 8, and 10 days.

### 2.6. Physicochemical and Sensory Properties of Meat Patties

#### 2.6.1. Texture Analysis

Measurements of texture parameters were performed using a universal texture analyzer (Universal Testing Machine Instron 3343, Instron Engineering Group, High Wycombe, England) equipped with a 1 kN load cell.

For the texture profile analysis (TPA), raw and cooked samples of the meat patties (with dimensions of 2.0 × 2.0 × 2.0 cm) were compressed perpendicularly using a 50 mm diameter cylindrical probe. The testing conditions included two consecutive cycles of 70% compression with crosshead movement at a constant speed of 1 mm/s. Texture variables (hardness, cohesiveness, chewiness, and springiness) were also evaluated [24].

#### 2.6.2. Sensory Evaluation

The sensory panel for descriptive analysis consisted of eight assessors experienced in the sensory evaluation of meat and other products. Assessors were selected and trained according to the ISO 8586 standard. Sensory evaluations were performed using a standardized sensory–descriptive method. The sensory attributes of cooked-meat patty samples were analyzed. A structured numerical scale was used to evaluate the intensity of each attribute. The left side of the scale corresponding to the lowest intensity of the attribute was assigned a value of one, and the right side corresponding to the highest intensity was assigned a value of nine. All the sessions were conducted in a climate-controlled sensory-analysis laboratory equipped with individual booths. The assessors were instructed to clean their palates with water or warm, weak tea between evaluations of each sample. The order of sample presentation was randomized. A data collection system for the automatic acquisition of assessor scores and data analysis was used (FIZZ, Biosystems, France).

For sensory evaluation, freshly cooked patty samples were cooled to 65 °C. The samples were quartered, placed onto a serving tray, and served immediately to the panelists along with room-temperature water, black tea, and white bread for receptors neutralization. The assessors were instructed to clean their palates with water or tea between evaluations of each sample. The following sensory characteristics were assessed: overall odor, meaty odor, vegetal odor, non-typical odor, hardness, stringiness, chewiness, juiciness, mouth coating, overall taste, meaty taste, vegetal (characteristic for plant products) taste, cooked-meat taste, non-typical taste, and aftertaste.

The focus group (*n* = 8, age 25–55) evaluated the preliminary acceptability of the cooked-meat patty properties including odor, taste, texture, and overall acceptability, and discussed the main properties that possibly limit acceptability.

#### 2.6.3. Technological Properties

The water-holding properties of the meat patties were analyzed by calculating the cooking loss. The cooking loss of the patty samples during cooking was determined according to the methodology proposed by Kouba [25]. After weighing, the uncooked samples were cooked in a convection oven at 200 °C for 20 min. Subsequently, the samples were cooled to 25 °C and weighed again. Cooking loss was calculated as (initial weight-final weight)/(initial weight) × 100 and expressed as a percentage. The amount of moisture retained in the cooked product per 100 g of sample was expressed as the moisture retention value and calculated according to the equation reported by El-Magoli et al. [26]. Fat retention (representing the amount of fat retained in the product after cooking) was calculated according to the method described by Murphy et al. [27].

### 2.7. Statistical Analysis

All measurements were performed at least three times and IBM SPSS^©^ Statistics (v.25, IBM, 2017) was used for data analysis. A one-way analysis of variance (ANOVA) was performed to evaluate statistical significance (*p* < 0.05) among the formulations. Multiple comparisons of means were performed using the Duncan test to determine significance (*p* < 0.05) among the formulations. The values reported in the tables represent mean values.

## 3. Results and Discussions

### 3.1. Properties of Oleogels

Changes in the composition of fatty acids are indicative of oxidative stability and the nutritional attributes of oils and fats. Oils constituting more unsaturated fatty acids than saturated fatty acids are easily oxidized as the unsaturated fatty acid components have elevated amounts of double bonds, which decrease oxidative stability [28].

The fatty acid composition of the oleogel oil is shown in Table 1. The hemp oil (control) and oleogels contained similar polyunsaturated fatty acids (PUFAs), saturated fatty acids (SFAs), and monounsaturated fatty acids (MUFAs). In quantitative terms, α-linolenic acid (C18:3) and linoleic acid (C18:2) were the main fatty acids present in the hemp oil and the oleogels (approximately 93%). The initial saturated fatty acid (SFA) content in hemp oil was low (9.22%), with monounsaturated fatty acids (MUFAs) having a content level of 10.28% and polyunsaturated fatty acids (PUFAs) reaching a content level of 80.47%. The SFA and MUFA contents decreased during the storage period in all samples. The PUFA concentrations increased in all samples from day 1 until the end of the storage period. The Omega 6/3 ratio of the hemp-seed-oil samples decreased slightly during the storage period.

The addition of candelilla and rice bran waxes to hemp seed oil may be a feasible and pragmatic approach to protect oil from oxidation during storage. Moreover, using natural waxes may provide a novel opportunity for producers and manufacturers to preserve the unsaturated fatty acids in oils. This approach can be further utilized to develop oleogels derived from candelilla and rice bran waxes to ensure the preservation of vegetable oils.

The oxidative stability results of the hemp seed oil and oleogels are presented in Table 2. The oxidative stability index (OSI), expressed as the induction period, shows the relative resistance of fats and oils to oxidation. The induction period (IP) value for the hemp-oil sample (control) was 3.10 h. The results revealed that hemp oil exhibited a shorter (*p* < 0.05) induction period than the oleogels. A similar tendency was determined for an oleogel made from soybean oil [29]. This indicates that natural waxes have a positive effect on preventing the oxidation of hemp oil.

The oleogels with rice wax (O-R5 and O-R7) exhibited significantly higher induction period values than those produced with candelilla wax (approximately 8%). However, it was observed that increasing the concentration of waxes in the oleogel shortens the induction period in both oleogel matrices.

These results are in agreement with the tendency determined for rapeseed oil, in which the stability of the oleogel depends on the properties and fatty acid composition of the oleogelator used [30].

Among different natural waxes, candelilla wax and rice bran waxes have been found to possess powerful gelling capabilities [31] which can decrease oxidation rates. This fact is beneficial to the nutritional quality of the oleogels, considering the high content of unsaturated fatty acids and the oxidation rate of the oil. Changes in the chemical stability of hemp oil and oleogels during storage at room temperature for 50 days are presented in Table 2.

A higher peroxide value indicates lower oxidative stability. The peroxide value of hemp oil gradually increased during storage; however, a significant decrease was observed at the end of the storage period. The same pattern of change was observed for the peroxide values of the oleogels. The peroxide value of hemp oil increased at a faster rate during storage and reached a higher value than that of the oleogels.

A high temperature (100 °C) was used during oleogel preparation, which could have a negative effect on the oxidation of the oleogels. For this reason, oleogel samples sometimes exhibit higher peroxide values than fresh oil, as has been found for canola oil (Lim, 2017). However, this tendency has not been observed for flaxseed-oil-based oleogels [32]. An analysis of the oleogels prepared in our study did not reveal significant differences in the peroxide values of the hemp oil and oleogels produced with the hemp oil.

The results suggested that, as the oleogels became harder their peroxide value lowered. This situation may arise because of the restriction of oil mobility and because migration via organogelation was effective in retarding oil oxidation during storage [33].

In our study, the oleogels revealed similar oxidation patterns to those of the control hemp-oil samples observed over the storage period, thus confirming the effects of several oxidation products provided by the mixture of waxes and hemp oil mixes. The peroxide values of all oleogel samples on day 20 were lower than that of the control hemp oil sample. The peroxide values of all samples reached their peaks at 25 °C on day 33.

Our findings are congruent with the results of other research groups, revealing a slower increase in peroxide value in oleogels prepared using rice bran wax and candelilla wax than in control oil [34]. On the other hand, the olive-oil-candelilla-wax oleogel demonstrated a higher peroxide value than stack oil at 20 °C [35]. The reasons for such a variation in peroxide value and rate of change could be attributed to the differences in experimental conditions such as sample preparation, proportion of surface area to the bulk of samples, storage temperature of samples, parameters of technological process, etc.

Candelilla and rice bran waxes demonstrated significant potential in delaying the time taken to attain peak peroxide value for the first 33 days of storage when compared to hemp oil. Plant compounds reduce lipid oxidation because of their radical scavenging capacity. Phenolic structures may delay the onset of oxidation due to the decline of hydroperoxides. Several phenolic compounds have distinct capabilities for postponing lipid oxidation. These different effects are traditionally explained by the diversity in their structure [36].

Free fatty acids (FFA) are formed by the hydrolysis of ester bonds in triglycerides by certain lipases. Our results showed that the acidity values (AV) and FFA content of the hemp-oil samples increased at the end of their storage period. Notably, the AV and FFA values of hemp oil were significantly higher than those of the oleogels.

There was a steady rise in the FFA content of the oleogel samples as well as in the control oil sample by day 33. The FFA content of the hemp-oil samples was higher than that of the oleogels.

On day 1, the acidity values of the oleogels were similar to those of the hemp-seed-oil samples. However, on day 33, the acidity of the hemp-oil samples reached 3.44 KOH/g oil, while for oleogels this value was significantly lower (Table 2). When compared to the control oil samples, the acidity values increased slightly until their peak point during storage. At the end of the storage period, the acidity values of rice-bran-wax oleogel samples were calculated to be 1.86 KOH/g oil for sample O-R5 and 2.00 KOH/g oil for sample O-R7. The acidity values measured for candelilla-wax oleogel samples were 1.96 KOH/g oil for sample O-C3 and 2.10 KOH/g oil for sample O-C7 mg KOH/g oil.

The high concentration of wax in hemp oil provides a higher degree of firmness, denser crystal networks, and higher melting points for oleogels’ physical properties [31]. To understand the effect of wax concentration on oil preservation, oleogels produced with two different concentrations of hemp oil were analyzed. The contents of candelilla and rice bran waxes did not affect the initial peroxide values. After six days of storage at 25 °C, the oleogel sample OR5 showed a lower PV than the remaining oleogel samples. Our results indicate that higher wax concentrations do not provide enhanced protection from oxidation of hemp oil.

The results of the conjugated diene (CD) value estimation showed that the oleogels exhibited a similar behavior (Table 2) to that of hemp oil.

The study results also indicate that increasing the concentration of rice bran wax may have a reverse effect on the oxidation protection of hemp oil. This may be attributed to the pro-oxidant effect of the rice bran wax. Interestingly, the peroxide values of the oleogel samples prepared on day six did not demonstrate significant pro-oxidant action, perhaps because of the shorter incubation/storage period, which was insufficient to display the desired effects. Similarly, sample OC3 showed a longer induction period (*p* < 0.05) at 3.65 ± 0.02 h when compared to sample OC7 with an incubation time of 3.45 h. This supports our conclusion that a higher rice-bran-wax content may have a pro-oxidant effect on hemp seed oil.

### 3.2. Properties of Meat Patties

#### 3.2.1. Malondialdehyde Value

Malondialdehyde (MDA) is one of the most toxic byproducts of changes in unsaturated fatty acids because it has cytotoxic and mutagenic properties owing to its ability to bind to nucleic acids and proteins, specifically to -NH2 and -SH groups, making it a tertiary lipid-oxidation product. MDA induces oxidative stress. Food preservation and cooling have a prohibitive impact on lipid oxidation [37]. We measured the MDA content of uncooked beef meat during storage at −1 °C.

**Figure 1:**
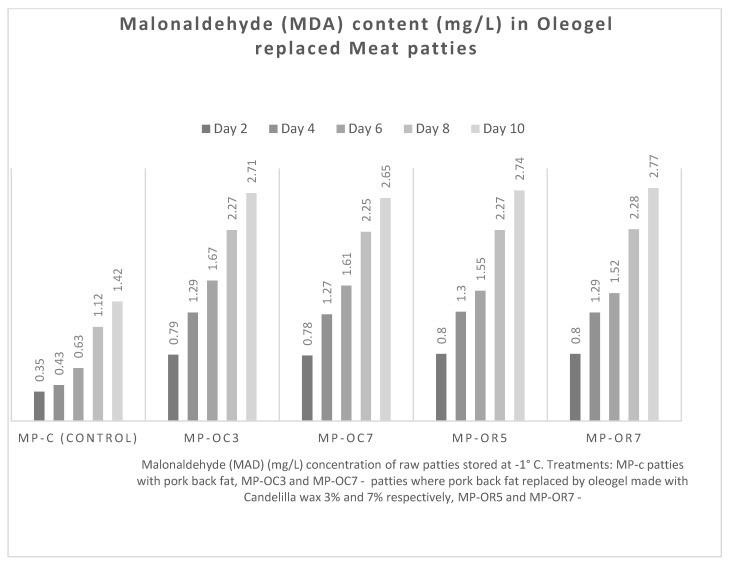
Concentration of malonaldehyde (MAD) of raw burgers during storage 2, 4, 6, 8, 10 days.

The results of MDA content (Figure 1) indicate that uncooked patties with pork back-fat were the most resistant and had lower oxidation levels (*p* < 0.05) compared to those of meat patties made with an oleogel during storage at −1 °C. For example, the MDA value of the control sample on day two was 0.35 mg/L (MDA). By day ten, the MDA value of this sample had reached 1.42 mg/L.

All samples of meat patties with oleogels had higher MDA values (MDA value–0.78 mg/L) than the control sample when the initial MDA value was approximately 0.35 mg/L. This contributed to elevations of MDA (from approx. 2.71 to 2.77 mg/L) during storage in all the meat patties with oleogels. However, no significant differences were determined in MDA level between different meat patties with oleogels at the end of the storage period. A higher level of fat oxidation can be explained through the higher quantity of polyunsaturated fatty acid (PUFA), which is a characteristic of hemp oil.

However, some studies have reported that meat batters in which pork back fat was replaced with a soybean-oil oleogel made with a mixture of cellulose and cellulose derivatives had better oxidative stability than the control sample containing pork fat [29].

#### 3.2.2. Proximate Composition and Technological Properties

The proximate compositions of meat patties with pork back fat (MP-C, control) samples and meat patties with oleogels are presented in Table 3. All processing formulas had a steady meat mass of 79%, and we intended to aim for a lipid content of approximately 20–21% in the most recent product. After the measurement of analytical values, all samples were within the expected scale. The moisture content of the uncooked- and cooked-meat patty samples was similar. The ash content of meat patties made with pork fat had the highest value at 2.45%, which was significantly higher (*p* < 0.05) than the ash content of meat patties made with oleogels. However, the opposite pattern was observed after cooking (Table 3). The highest fat loss during cooking was observed in the OC3-fortified meat patty. However, there was a difference (*p* < 0.05) in the protein content of the formulations, even though this change was only an increment of 2% (from 25% to 27%). The ash content was also significantly different (*p* < 0.05) between the formulations.

The cooking loss of the control sample was 20% lower than that of the modified meat patties containing oleogels. In addition to lower fat levels, the higher cooking loss in meat patties containing oleogels may occur because of the lower thermal stability of the prepared oleogels. This may be a driving factor for higher fat loss during cooking. Fat retention in meat matrices after thermal processing is indispensable for guaranteeing the high sensory quality of the products [38].

The most common alternative to reducing fat content in meat patties is to replace lard with lean meat or water, but these modifications usually decrease the moisture retention capacity of the matrices [39]. Our results indicate that all the prepared oleogels showed no significant differences in moisture retention. Cooking loss for meat patties containing oleogels was noticeably higher than that of control meat patties, and fat retention was much lower. The lowest fat-retention rate was observed in patties with an oleogel sample (MP-OC3) prepared with 3% candelilla wax, thus supporting the conclusion that lipid reformulation is effective in improving important technological properties of the matrices and ensuring a healthier lipid profile.

#### 3.2.3. Fatty Acid Profile

The fatty acid composition of the uncooked and cooked meat patties is presented in Table 4. Samples with pork back fat had very different fatty acid compositions compared to the samples made with oleogels. This could be attributed to the different types of oils present in the samples. The Omega 6/3 content of the control sample containing pork fat was significantly higher than that of the samples fortified with oleogels. The SFA content in the control sample was more than two times higher than the SFA content in the oleogel samples. The SFA content of meat matrices containing pork meat was approximately 40%. The PUFA content of meat matrices containing oleogels ranged from 68% to 80%, which was significantly higher than that of samples with meat patties containing pork back fat. The fatty-acid-composition results revealed that patties containing oleogels are a potentially viable source of PUFAs. However, samples containing pork fat contained higher amounts of SFAs than the majority of fatty acids. Owing to the composition of fatty acids, meat patties with pork back fat might be more stable against oxidation. However, samples containing hemp-oil oleogels could provide more benefits to cardiovascular health upon consumption.

#### 3.2.4. Physicochemical and Sensory Properties

A texture profile analysis of meat patties revealed a significant effect of the replacement of pork back fat with oleogels (Table 5). The use of candelilla wax for oleogel structuring resulted in harder-cooked products, whereas in uncooked samples the effect depended on the amount of added candelilla wax.

A use of 3% candelilla wax in oleogels resulted in a lower hardness value than the control sample, while a sample using 7% of candelilla wax in oleogels had a higher hardness value than the control. In general, MP-OC3 and MP-OC7 uncooked samples scored lower in gumminess and springiness parameters than the control sample. Cooked samples with oleogels developed from candelilla wax were harder and had higher gumminess than the control sample, but their cohesiveness and springiness were similar to those of the control sample.

When a 7% of rice bran wax concentration was used to develop oleogels, TPA values for uncooked samples were similar to those of the control sample. The uncooked MM-OR5 sample had lower hardness, gumminess, and springiness values than the control sample. However, the situation was different for the cooked samples. In general, the control sample had lower hardness and gumminess than the MM-OR5 and MM-OR7 samples.

This significant change in texture parameters during cooking could possibly be explained by different cooking losses between control samples with fat and samples with part of the fat replaced with oleogels. A similar tendency has been observed in meat emulsions prepared with oleogels made from canola oil [40].

The sensory profiles of cooked patties (Table 6) revealed that all samples had similar odor intensity, but samples with oleogels had a less pronounced odor specific to meat. The non-meat odor was more intense in samples with oleogels developed using rice bran wax than in the control or samples with oleogels developed using candelilla wax. The effect of candelilla or rice bran wax concentration used for oleogel production was not detected; however, for most of the odor or taste properties, the replacement of pork fat with oleogels had a significant effect.

The hardness values of samples MM-OR5 and MM-OR7 were higher than those of the other samples, which is in agreement with the instrumentally measured texture profile data (Table 5).

Control samples (formulated only with pork back fat) had higher juiciness values (*p* < 0.001) and were softer (*p* < 0.05) than oleogel samples. During the chewing process of patties with oleogels, some level of greasiness was perceived in the mouth which was not observed in the control sample. This could be explained by the lower water–oil binding capacity of the patties made with oleogels, as determined by cooking loss.

Sensory data showed a clear tendency for replacement of pork back fat in meat patties containing oleogels developed using candelilla wax, which decreased the intensity of meaty odor and taste. The same effect was observed for the juiciness of the patties. However, in these samples there was a perceived odor and taste that is non-typical for meat. Some greasiness was also observed during the in-mouth feel assessment. These data are in contrast to data observed on frankfurters, where samples with oleogels had higher juiciness than samples made with pork fat [41].

Oleogels developed using rice bran wax and used as a replacement for pork back fat in meat patties resulted in a lower intensity of meat odor and taste, higher hardness and greasiness, and lower juiciness when compared to the control sample.

Some studies have shown that the replacement of fat with soybean-oil oleogels had no effect on the sensory acceptance of meat batters [29], even when significant changes in the texture properties of samples were observed. In contrast, when sesame-oil oleogels replaced 50% of the fat in beef burgers, the texture parameters of the samples did not change but the samples were found to be more acceptable than the control samples, possibly because of the specific taste and odor of sesame oil [42]

## 4. Conclusions

Our results showed that candelilla and rice bran waxes used to develop oleogels based on hemp seed oil increased its oxidative stability when compared to that of fresh hemp seed oil. A texture analysis revealed that oleogels were able to match pork back fat with some textural properties, such as spreadability and adhesiveness. Cooking losses were lower for control samples, whereas cooking losses were increased by 20% for samples containing oleogels. A sensory analysis of cooked patties revealed that it is possible to replace pork back fat with prepared oleogels without any negative effects on patty odor properties. However, the control samples (formulated only with pork fat) rated higher than the meat patties fortified with oleogels for parameters such as in-mouth feel and juiciness. The control samples exhibited intense flavor properties. This could be explained by the lower water–oil binding capacity of patties made with oleogels, as determined by cooking loss. Our results support wax-based oleogels developed from candelilla and rice bran as potential pork back fat substitutes in meat patty matrices. However, methods to improve meat patty oil-binding capacity with the aim of maintaining juiciness typical of conventional meat patties should be developed.

## Figures and Tables

**Table 1:** Fatty acid profile of the hemp oil and oleogels during storage.

Fatty Acid	Hemp Oil (Control)			O-C3			O-C7			O-R5			O-R7		
	Duration of Storage, Days														
	D1	D15	D35	D1	D15	D35	D1	D15	D35	D1	D15	D35	D1	D15	D35
C16:0	5.60	5.31	5.35	5.42	5.29	5.39	5.46	5.40	5.37	5.70	5.42	5.33	5.66	5.32	5.31
C18:0	2.29	2.13	2.09	2.14	2.15	2.10	2.16	2.14	2.10	2.29	2.14	2.11	2.24	2.12	2.07
C18:1	9.77	9.18	9.27	9.43	9.15	9.34	9.49	9.40	9.30	9.94	9.25	9.25	9.69	9.16	9.17
C18:2cis	56.81	57.40	56.55	57.11	57.69	56.94	56.85	57.14	56.95	56.43	57.05	57.31	56.72	57.44	57.12
C18:3a	18.16	18.53	18.35	18.49	18.26	18.70	18.47	18.36	18.71	18.22	18.34	18.48	18.30	18.42	18.81
C18:3	4.12	4.24	4.27	4.24	4.24	4.38	4.33	4.23	4.40	4.21	4.21	4.27	4.26	4.25	4.33
C20:2	1.35	1.38	1.34	1.32	1.39	1.32	1.35	1.37	1.33	1.33	1.36	1.36	1.34	1.38	1.38
Trans Total	0.02	0.01	0.01	0.02	0.00	0.01	0.02	0.12	0.01	0.02	0.11	0.01	0.02	0.01	0.01
SFAs	9.22	8.75	8.96	8.85	8.78	8.78	8.91	8.85	8.74	9.29	9.16	8.77	9.13	9.16	8.67
MUFAs	10.28	9.65	9.98	9.91	9.61	9.82	9.97	9.89	9.80	10.47	9.73	9.73	10.19	9.64	9.64
PUFAs	80.47	81.59	81.45	81.21	81.61	81.38	81.08	81.13	81.44	80.22	80.99	81.47	80.65	81.53	81.67
n-3 PUFA	18.20	18.57	18.93	18.54	18.29	18.74	18.55	18.40	18.75	18.25	18.38	18.52	18.34	18.42	18.84
n-6 PUFA	62.28	63.02	62.52	62.67	63.32	62.64	62.54	62.74	62.69	61.97	62.61	62.95	62.32	63.07	62.83
n-9 PUFA	10.14	9.54	9.81	9.79	9.51	9.72	9.85	9.77	9.70	10.31	9.61	9.62	10.06	9.53	9.53
Omega 6/3	3.422	3.394	3.303	3.380	3.462	3.343	3.371	3.410	3.343	3.396	3.406	3.399	3.398	3.424	3.335

Treatments: O-C3 and O-C7—oleogels prepared from hemp oil with candelilla wax concentrations of 3% and 7%, respectively; O-R5 and O-R7—oleogels prepared from hemp oil with rice wax concentrations of 5% and 7%, respectively.

**Table 2:** Values of parameters showing chemical stability of the hemp oil and oleogels.

Parameter	Storage Duration, Days	Hemp Oil (Control)	O-C3	O-C7	O-R5	O-R7
Induction Period (hours)	-	3.1 ^a^	3.65 ^b^	3.45 ^b^	3.95 ^c^	3.75 ^c^
Peroxide Value (meq/kg)	1	2.18	2.17	2.31	2.19	2.18
	6	7.73 ^b^	6.92 ^ab^	7.10 ^ab^	6.30 ^a^	6.87 ^ab^
	12	10.70 ^b^	8.65 ^a^	9.30 ^a^	8.10 ^a^	8.22 ^a^
	20	13.51 ^b^	11.34 ^a^	12.47 ^a^	11.96 ^a^	10.24 ^a^
	33	16.76 ^b^	13.85 ^a^	14.10 ^a^	12.44 ^a^	13.75 ^a^
	50	13.72	12.31	13.80	13.34	12.78
Acidity Value (KOH/g oil)	1	0.55	0.52	0.51	0.50	0.53
	6	0.92 ^b^	0.64 ^a^	0.68 ^a^	0.62 ^a^	0.63 ^a^
	12	1.31 ^b^	0.93 ^a^	0.91 ^a^	0.84 ^a^	0.87 ^a^
	20	2.69 ^b^	1.70 ^a^	1.77 ^a^	1.92 ^a^	1.95 ^a^
	33	3.44 ^b^	2.55 ^a^	2.85 ^a^	2.96 ^a^	3.10 ^a^
	50	2.12	1.96	2.10	1.86	2.00
Free Fatty Acids Content (% oleic acid)	1	0.22	0.20	0.21	0.22	0.21
	6	0.41	0.35	0.37	0.34	0.38
	12	0.52	0.42	0.46	0.43	0.46
	20	0.74 ^b^	0.48 ^a^	0.51 ^a^	0.47 ^a^	0.55 ^a^
	33	1.22 ^b^	0.91 ^a^	0.98 ^a^	0.83 ^a^	0.88 ^a^
	50	0.98	0.95	0.94	0.99	1.00
Conjugated Diene Value	1	1.33	1.34	1.30	1.39	1.15
	6	4.55	5.35	6.96	5.51	5.42
	12	6.46	6.98	7.86	6.94	6.74
	20	8.28	9.42	9.51	9.24	9.14
	33	9.24 ^a^	11.69 ^b^	12.32 ^b^	12.62 ^b^	11.09 ^b^
	50	12.75	14.15	13.08	14.26	13.54

For each parameter, different letters in the same column indicate significant differences (*p* < 0.05). Treatments: O-C3 and O-C7—oleogels prepared from hemp oil with 3% and 7% candelilla wax concentrations, respectively, and O-R5 and O-R7—oleogels prepared from hemp oil with 5% and 7% rice wax concentrations, respectively.

**Table 3:** Proximate analysis and technological properties of uncooked and cooked meat patties.

	MP-C (Control)	MP-OC3	MP-OC7	MP-OR5	MP-OR7
Moisture (%)					
Uncooked	62.00	58.70	60.10	60.10	60.00
Cooked	50.00	53.90	50.20	49.00	51.50
Ash (%)					
Uncooked	2.45 ^b^	1.93 ^a^	1.99 ^a^	1.94 ^a^	1.93 ^a^
Cooked	2.00 ^a^	2.36 ^b^	2.44 ^b^	2.28 ^b^	2.74 ^b^
Protein (%)					
Uncooked	18.30	18.20	17.80	18.10	18.10
Cooked	25.00	27.30	26.60	26.90	27.00
Cooking loss%	24.48 ^a^	38.58 ^c^	33.69 ^b^	34.16 ^b^	40.35 ^c^
Moisture retention (%)	35.76 ^b^	33.11 ^a^	33.29 ^a^	32.26 ^a^	30.72 ^a^
Fat retention (%)	93.27 ^c^	43.54 ^a^	62.10 ^b^	68.17 ^b^	58.01 ^b^

Treatments: MP-C—control meat patties in which pork back fat was used as fat; MP-OC3 and MP-OC7—meat patties in which pork back fat was replaced by an oleogel prepared from hemp oil with candelilla wax concentrations of 3% and 7%, respectively; MP-OR5 and MP-OR7—meat patties in which pork back fat was replaced by oleogel prepared from hemp oil with rice wax concentrations of 5% and 7%, respectively. For each parameter, different letters in the same row indicate significant differences (*p* < 0.05) based on ANOVA.

**Table 4:** Fatty acid composition of uncooked and cooked meat patties.

	Uncooked Samples					Cooked Samples				
	MP-C (Control)	MP-OC3	MP-OC7	MP-OR5	MP-OR7	MP-C (Control)	MP-OC3	MP-OC7	MP-OR5	MP-OR7
C14:0	1.19	0.12	0.48	0.13	0.17	1.62	0.20	0.23	0.20	0.17
C16:1	1.83	0.18	0.53	0.21	0.23	2.17	0.31	0.35	0.27	0.26
C16:0	24.96	6.25	9.30	6.31	6.77	25.35	7.26	7.43	7.01	6.92
C18:0	13.02	2.41	4.13	2.50	2.72	13.46	2.93	2.98	2.81	2.74
C18:1	42.27	9.00	12.38	9.81	9.70	41.13	10.66	10.83	10.54	10.26
C18:2cis	12.52	55.95	48.28	55.80	55.25	11.30	53.50	52.27	54.29	54.70
C18:3a	0.78	18.13	16.24	18.14	18.09	0.89	17.28	16.93	17.68	17.93
C18:3 g	NA	3.77	3.34	3.63	3.68	0.04	3.55	3.45	3.54	3.63
C20:2	0.46	1.29	1.10	1.21	1.24	0.43	1.19	1.14	1.18	1.21
Trans Total	0.23	0.03	0.26	0.06	0.06	0.58	0.09	0.10	0.08	0.07
SFAs	40.08	10.19	17.35	10.52	11.11	41.54	11.82	14.14	11.63	11.26
MUFAs	45.53	9.58	13.43	10.51	10.48	44.72	11.52	11.66	11.33	11.01
PUFAs	14.15	80.19	68.96	78.89	78.34	13.14	76.57	74.08	76.95	77.66
n-3 PUFA	1.05	19.19	16.24	18.25	18.18	1.25	18.33	16.93	17.96	18.12
n-6 PUFA	13.10	61.00	52.72	60.65	60.16	11.89	58.24	57.16	59.00	59.54
n-9 PUFA	43.38	9.34	12.70	10.23	10.17	42.02	11.10	11.19	10.98	10.65
Omega 6/3	12.48	3.18	3.25	3.32	3.31	9.51	3.18	3.38	3.29	3.29

Treatments: MP-C control meat patties in which pork back fat was used as fat; MP-OC3 and MP-OC7—meat patties in which pork back fat was replaced by an oleogel prepared from hemp oil with Candelilla wax concentrations of 3% and 7%, respectively; MP-OR5 and MP-OR7—meat patties in which pork back fat was replaced by an oleogel prepared from hemp oil with rice wax concentrations of 5% and 7%, respectively.

**Table 5:** Texture parameters of uncooked and cooked meat patties with different oleogels.

		Sample				
Matrices	Texture Parameter	MP-C (Control)	MP-OC3	MP-OC7	MP-OR5	MP-OR7
Raw	Hardness (N)	7.73 ^b^	3.97 ^a^	10.38 ^c^	4.49 ^a^	7.60 ^b^
	Cohesiveness (Ratio)	0.35 ^b^	0.28 ^ab^	0.23 ^a^	0.33 ^b^	0.32 ^b^
	Gumminess	2.77 ^c^	1.09 ^a^	2.32 ^b^	1.44 ^b^	2.39 ^c^
	Springiness	2.66 ^b^	1.90 ^a^	1.68 ^a^	1.85 ^a^	1.89 ^a^
Cooked	Hardness N. (N)	52.05 ^a^	91.26 ^b^	90.43 ^b^	167.06 ^c^	169.96 ^c^
	Cohesiveness (Ratio)	0.56 ^ab^	0.52 ^a^	0.62 ^b^	0.51 ^a^	0.48 ^a^
	Chewiness	30.83 ^a^	48.40 ^b^	55.10 ^b^	83.85 ^c^	96.11 ^d^
	Springiness	4.69 ^a^	4.76 ^a^	5.22 ^b^	5.11 ^ab^	4.47 ^a^

^a, b, c^—For each parameter, different letters in same row indicate significant differences (*p* < 0.05) based on Duncan test. Treatments: MP-C control meat patties in which pork back fat used as fat; MP-OC3 and MP-OC7—meat patties in which pork back fat was replaced by an oleogel prepared from hemp oil with candelilla wax concentrations of 3% and 7%, respectively; MP-OR5 and MP-OR7—meat patties in which pork back fat was replaced by an oleogel prepared from hemp oil with rice wax concentrations of 5% and 7%, respectively.

**Table 6:** Mean scores of the sensory attributes, intensity values of cooked meat patties.

		Sample				
Sensory Attribute		MP-C (Control)	MP-OC3	MP-OC7	MP-OR5	MP-OR7
Odor	Overall intensity	7.25	5.88	6.38	7.25	6.25
	Meaty	7.25 ^c^	6.00 ^b^	6.25 ^b^	5.63 ^a^	4.75 ^a^
	Vegetal	1.00 ^a^	3.50 ^b^	2.50 ^b^	4.00 ^c^	4.63 ^c^
	Non-typical	1.13 ^a^	2.63 ^b^	2.38 ^b^	2.25 ^b^	3.50 ^b^
Texture	Hardness	4.13 ^a^	3.88 ^a^	3.75 ^a^	5.13 ^b^	4.88 ^b^
	Springiness	5.88	4.88	5.38	5.13	4.88
	Chewiness	5.50	5.38	4.88	4.88	5.38
	Greasiness	1.50 ^a^	5.75 ^b^	6.28 ^c^	4.50 ^b^	5.63 ^b^
	Juiciness	4.88 ^c^	3.75 ^b^	4.18 ^b^	2.88 ^a^	2.63 ^a^
	Mouth coating	4.50	3.50	4.38	2.88	3.63
Taste	Overall intensity	6.75	6.25	6.75	7.00	6.38
	Meaty taste	6.88 ^b^	5.38 ^a^	5.88 ^a^	4.88 ^a^	5.50 ^a^
	Vegetal	1.38 ^a^	3.88 ^b^	4.00 ^b^	5.13 ^c^	5.25 ^c^
	Aftertaste	2.13	2.88	3.50	2.63	4.00
	Non-typical	1.00 ^a^	1.80 ^b^	1.80 ^b^	1.80 ^b^	1.90 ^b^

^a, b, c^: For each parameter, different letters in the same row indicate significant differences (*p* < 0.05) based on Duncan’s test. Treatments: MP-C—control meat patties in which pork back fat was used as fat; MP-OC3 and MP-OC7—meat patties in which pork back fat was replaced by an oleogel prepared from hemp oil with candelilla wax concentrations of 3% and 7%, respectively; MP-OR5 and MP-OR7—meat patties in which pork back fat was replaced by an oleogel prepared from hemp oil with rice wax concentrations of 5% and 7%, respectively.

## Data Availability

Data is contained within the article.
